# Evaluating the accessibility of public service facilities to tourists and residents in island destinations: Evidence from the Changhai County

**DOI:** 10.3389/fpubh.2022.1090341

**Published:** 2023-01-09

**Authors:** Xiaoling Zheng, Dong Zhao

**Affiliations:** ^1^Key Laboratory of Watershed Geographic Sciences, Nanjing Institute of Geography and Limnology, Chinese Academy of Sciences, Nanjing, China; ^2^University of Chinese Academy of Sciences, Beijing, China; ^3^Department of Natural Resources, Science and Technology and Foreign Exchange and Cooperation Division, Nanjing, China

**Keywords:** island tourism, public service facilities, tourists and residents, urban network analysis (UNA), Changhai County

## Abstract

With the increasing diversity of social groups, public service facilities need to meet the diverse needs of different groups. However, there is still a lack of in-depth research evaluating urban public service facilities for diverse groups. Therefore, this paper use Kernel density spatial analysis method to delimits the research area based on data on the temporal and spatial behavior of islanders and tourists, and use urban network analysis (UNA) method to evaluates the public service facilities of the spatially overlapping area from the aspects of facility accessibility and availability. The study shows that (1) the spatial dislocation between facilities and residential places is serious, which leads to redundant construction or a lack of configuration of facilities in some areas. (2) The public service facilities in some areas can be used by tourists and residents to a similar degree, the number of facilities accessible to residents and tourists within a certain distance is not much different, and the configuration of facilities is relatively reasonable. (3) The overall configuration of infrastructure is biased toward residents, but the configuration of facilities in some areas can also reflect group fairness. The results indicate that the public facilities have a tendency to serve residents, and the results can give some suggestions for public facilities configuration to build a human-oriented island.

## 1. Introduction

As a carrier of public services for residents' daily lives (1), public service facilities should be guided by the principle of fairness so that all residents within the scope of facility services can enjoy public services equally. Public service facilities include education, medical care, culture and sports, commercial services, municipal public utilities and other facilities (2), and they are the material guarantee for improving the quality of life of residents and have strong external effects. In the context of the transformation of the main social contradictions in the new era of China, residents' demand for public service facilities is no longer limited to survival needs. Rather, it has gradually turned to development and enjoyment needs (3). Therefore, meeting the diverse needs of residents and realizing the equalization and precise allocation of public service facilities are one of the requirements of new urbanization construction (4). Furthermore, with the development of cities or tourist destinations, the heterogeneity of social groups increases, and societies become more diverse. The configuration of public service facilities should be more in line with individual needs and should gradually be realized from regional equality to spatial equality and social equality, rooted in the people-oriented planning concept of new urbanization construction.

In recent years, with the development of tourism, islands or other place have gradually become the consumption space of urban society (5). Many scholars attach importance to island tourism (6), ice-snow tourism (7), rural tourism (8) and ecotourism research (9). The island tourism research, on the one hand, scholars at home and abroad focuses on tourism product development and marketing (10, 11). On the other hand, pay attention to the relationship between island tourism and ecological environment, society, economy, etc. Including the impact of economic development on tourism (12), and also include the impact of island tourism on the ecological environment (13), society and economy (14–16), such as the impact of tourists' behavior on the island environment (17), and the impact of island facilities on tourism activities. For example, the authors analyze that the improvement of island public service facilities can significantly increase the number of tourists (18). In general, there are relatively few studies on island public facilities at present, but from the existing studies, improving public service facilities is more important for island tourism, and many islands in the world are almost facing the same problem, that is, tourists are far higher than local residents in the peak tourism season, leading to the island infrastructure can not meet the actual needs. For example, scholars have analyzed analyzed the problems of sewage facilities and proposal sustainable segment management strategies on the islands in Croatia (19). In fact, in tourism destinations, as the tourism space conflict between local residents and tourists about the possession of resources with the nature of public goods often occurs (20), island public service facilities also belong to resources with strong public attributes, so the research on the allocation of public service facilities for island tourism destinations is of great significance to alleviate tourism space conflict.

In fact, the research on public service facilities began as early as the second half of the 19th century. With the proposal of the location theory of public service facilities (21), scholars outside China carried out in-depth research on the location selection of public service facilities (22), facility accessibility (23), and spatial fairness and its social and economic benefits (24, 25). At the end of the 20th century, research on the configuration of public service facilities that was oriented toward determining the actual layout of a city became a research hot spot in China. Scholars first paid attention to the spatial layout and location of public service facilities (26) and then carried out research on the characteristics of the spatial layout and the accessibility of public service facilities (27–29), social differentiation and satisfaction with public service facilities (30, 31). With the development of geographic information system technology and attention to social equity, research on the accessibility of public service facilities has become a hot topic. Scholars outside China have carried out empirical research on the accessibility of schools and hospitals, the concept of accessibility (32, 33) and the influencing factors of accessibility (34, 35). Chinese scholars have mostly focused on cities, counties and other regions and conducted accessibility evaluation research on parks, green spaces, educational facilities, or medical facilities (36, 37). Based on existing studies, current research outside China has entered the stage of social equity, and most Chinese research is still in the stage of spatial equity. Scholars in China and elsewhere have conducted research on the accessibility of public service facilities and the fairness of facility allocation.

There have been many research results, but there are still shortcomings: ① From the perspective of research objects there are few studies on evaluating the accessibility of different types of public service facilities, such as shopping and catering facilities, and the research objects are mostly urban or rural residents. That is, the research objects are a single group or are based only on age, occupation, or income to divide people into different categories. The utilization of public service facilities in existing research changes little over the course of a year. There are few comparative studies on the fairness of different activity groups in tourist destinations. ② From the perspective of the research area studies take cities, counties, and streets as homogeneous units. However, the optimal allocation of public service facilities is affected by factors such as the area type, grade and population, and it needs to undergo different stages to achieve social equity and universal sharing. Therefore, under the guidance of the people-oriented principle, the optimal configuration of public service facilities should start from common needs and rigid needs with a high degree of urgency, then the local optimization of elastic needs, and finally individual needs (38). The existing research does not consider the priority of public service facilities configuration, which is inappropriate.

In summary, with the heterogeneity of social groups increasing, society become more diverse. This paper takes island tourism destination as the research object, because the tourist destinations have obvious slow and peak seasons, and there are obvious differences in the main activity groups during the year. Different from cities or villages, in tourist destinations, the configuration of public service facilities needs to not only consider the daily needs of local residents but also meet the needs of tourists. In addition, the large difference in the number of residents and tourists in tourist destinations has led to a surge in the demand for facilities during the peak tourist season. With the departure of tourists, the idleness of facilities has also become a problem that urgently needs to be solved. Therefore, for tourist destinations, the allocation of public service facilities needs to consider group fairness and allocation efficiency (39). Hence, assessing the allocation of infrastructure is conducive to meeting the needs of diverse activity groups, effectively responding to the daily needs of residents as well as the needs of tourists and improving the group equity and the efficiency of facility allocation as much as possible. Therefore, choosing the island tourism destination as the research area can better reveal the fairness and efficiency problems reflected in the public service facility configuration under the background of social group diversification, which is the first innovation of this paper. At the same time, this study can make suggestions on the allocation of public service facilities through the study of infrastructure evaluation, and will also provide experience for other regions similar to island tourism destinations. On the other hand, different from the existing research, this paper based on judging the overlapping area of the behavioral spaces of tourists and residents, take facilities with high common demand as the research object and evaluates facility accessibility, which is the configuration process of public service facilities from common needs to personalized needs, and this is the second innovation of this paper. The questions of interest are as follows: (1) What is the current spatial configuration of public service facilities in the study area? (2) What is the availability of catering and shopping facilities for residents and tourists? (3) What is the accessibility of shopping and catering facilities to residents and tourists? What is the difference? This study expects to propose suggestions on spatial optimization for this area.

## 2. Research materials and methodologies

### 2.1. Study area

The area of this study is Changhai County (38°55′-39°18′N, 122°13′-123°17′E), which is situated in northeastern China. It is a typical tourist destination with a clear off-peak season. The tourist season is from May to October every year. The main activity groups on the island include residents and tourists. November to April of the following year is the tourist off-season, tourists and related practitioners leave, and residents constitute the main activity group on the island. There is a large gap between the number of tourists and residents. According to the 2019 “Changhai Statistical Yearbook,” the total number of tourists in Changhai County reached 1.34 million, which was 19 times the total number of local residents in that year. There are obvious differences in the main activity groups in the case area over the course of a year, and the allocation of public service facilities should take into account the issue of group fairness and the issue of allocation efficiency.

**Figure 1:**
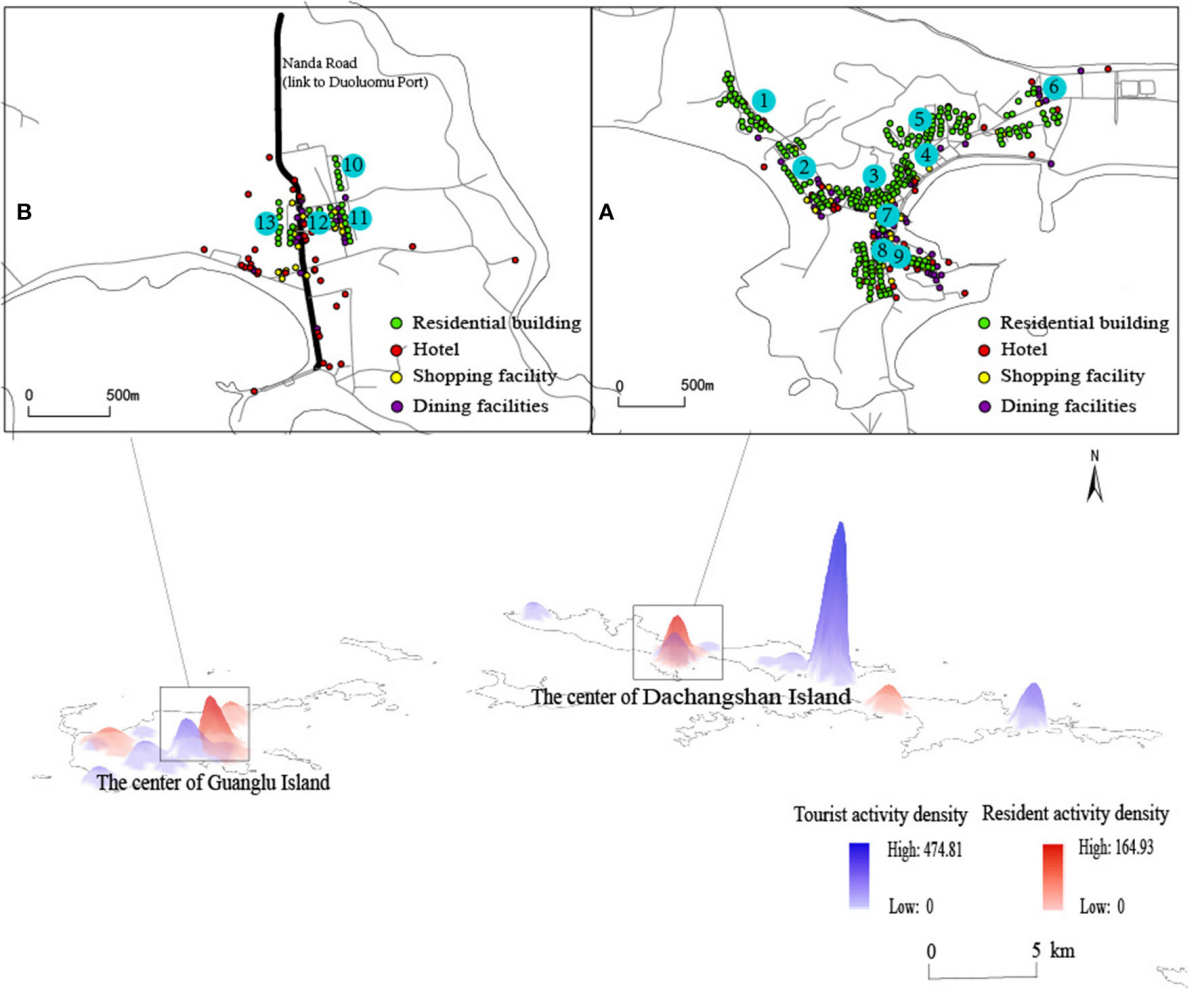
Location of the study area. **(A)** The center of the Dachangshan island. **(B)** The center of the Guanglu island. The numbers 1-13 represent the mainly places in the study area.

This paper uses activity log survey data from island residents and tourists in January and July 2019 and ArcGIS 10.2 software to perform kernel density estimation of the temporal and spatial behavioral activities of residents and tourists to show the spatial range of their behaviors. The behavioral space of residents represents the area where public service facilities may be used in the tourist off-season, and the behavioral space of tourists represents where public service facilities may be used in the peak season. The reason is that the peak season is from May to October every year, and the number of tourists far exceeds the number of residents. Therefore, the behavior space generated by tourist activities can represent the area where the public service facilities can be used during the peak season, while the tourism off-season is from November to April of the next year, and almost no tourists appear. The behavior space formed by residents' activities is the area where the public service facilities can be used during the off-season. As shown in Figure 1, most of the behavioral spaces of residents and tourists are scattered, and only two parts of the area overlap. Therefore, the overlapping area of the behavioral spaces of residents and tourists is the research area, and priority should be given to the optimal configuration of public service facilities in this area.

### 2.2. Data source and processing

#### 2.2.1. Questionnaire survey data

From January 24 to 27 and from July 27 to August 1, 2019, the research team conducted a questionnaire survey on the residents and tourists of Dachangshan Island, Xiaochangshan Island and Guanglu Island. The survey content included personal socioeconomic attributes, activity itineraries and other information. A total of 310 and 315 questionnaires were distributed to residents and tourists, respectively, and 285 and 303 were recovered, respectively.

#### 2.2.2. Data on public service facilities

**Figure 2:**
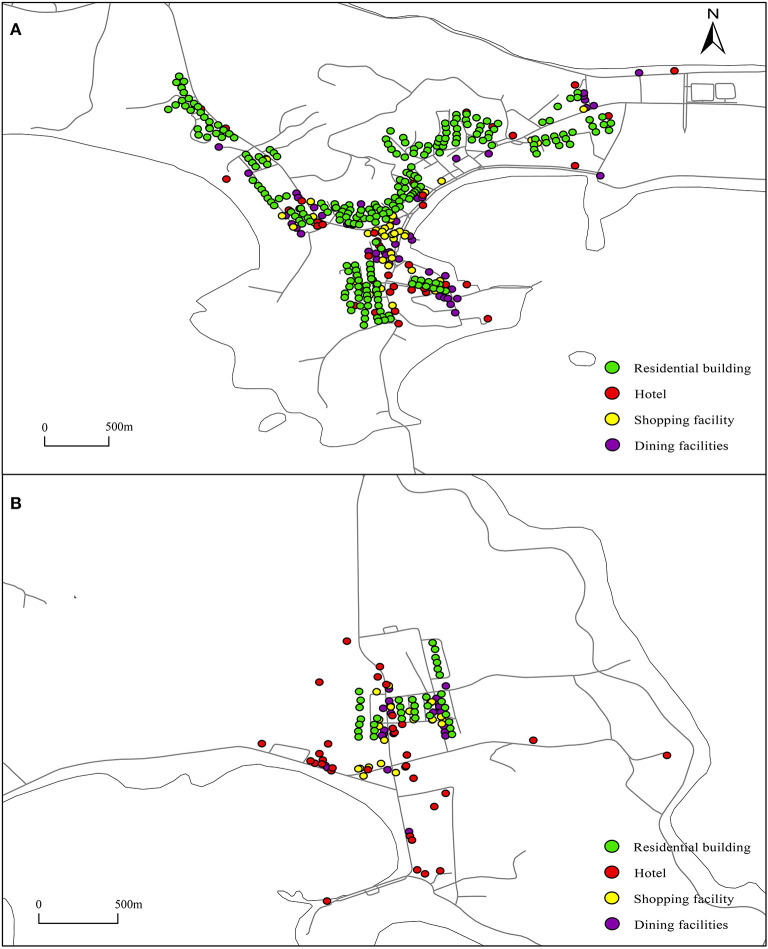
Distribution of facilities in the study area. **(A, B)** The results are counted according to the number of the facilities, including catering and shopping facilities, hotel and residential buildings.

The overlapping area of the behavioral spaces of residents and tourists is the space shared by the two groups. The degree of infrastructure sharing in the region is higher than that in other regions, but not all public service facilities in the region have the same degree of sharing. The degree of sharing of facilities that can meet some of the common needs of tourists and residents is the highest. In existing research on the degree of sharing, Ta and other scholars conducted research on the spatial isolation of Shanghai suburban residents by constructing the spatial sharing degree index (40). Sun and other scholars took the social life circle division as an example (41). In the former, the sharing index was determined by the number of different groups existing in the research area. The higher the number of groups is, the higher the sharing degree. In the latter, the research area was jointly utilized by multiple communities. Both studies conducted research from the perspective of spatial sharing but ignored the situation of facility sharing in the same space, that is, a situation where the same infrastructure is used by different groups. Tourists and residents are part of the unity of society and are natural persons. The tourism process includes activities such as eating, lodging, traveling, playing, shopping, etc. Eating is one of the common activities of tourists and residents. In addition, shopping facilities such as shops and convenience stores are shared infrastructures for residents and tourists. Then this paper selects catering facilities and shopping facilities with a high degree of sharing in the overlapping area as the research objects. Through the open platforms of Baidu Map and AutoNavi Map, the catering facilities, shopping facilities, tourist accommodation facilities such as fishing village restaurants and hotels, residential buildings and road network data of the study area were obtained. A total of 76 fishing village restaurants, 61 shopping facilities such as shops or supermarkets, 140 catering facilities, and 269 residential buildings were obtained. The distribution of facilities is shown in Figure 2. Area A is the center of Dachangshan Island, and area B is the center of Guanglu Island.

### 2.3. Methodologies

This paper installs the urban network analysis tool (Urban Network Analysis (UNA) Toolbox plug) in Rhino 6.13 software and uses the reach function of the tool for analysis. The advantage of this analysis tool is that actual roads are regarded as a network, a facility is regarded as a node, and, rather than using the activity radius as a circle to calculate the travel distance, the distance of the road network is used as the actual activity distance of an individual, which is closer to reality (42, 43). Through the reach index (Reach) in the UNA tool, the number of target points reachable from the starting point under the condition of the shortest path is calculated. In other words, the number of accessible facilities within a certain distance is calculated using residential building as the starting point. Additionally, the number of accessible residential buildings is calculated using the facilities as a starting point. The number of reachable target points is assigned as a weight to the starting point and exported to ArcGIS for spatial display. The specific formula is as follows:


(1) $$\begin{array}{l} {R\left[i\right]}^{r}=\sum_{j\in G-\left\{i\right\},d\left[i,j\right]\leq r}w\left[j\right]\ldots \ldots \end{array}$$

In the formula, *R*[*i*]^*r*^ represents the number from starting point i to destination j within distance r and G is the transportation network in the study area. *d*[*i, j*] represents the shortest distance from origin i to destination j. *w*[*j*] represents the weight of destination point j. Referring to existing research, the range of the community living circle is usually set as the range within 15 min that residents can reach by walking, that is, the range of 800 m from their homes (44). Meanwhile, 800 m corresponding to 10 min of walking is considered as an acceptable walking distance (45).Therefore, this paper sets distance r to 800 m. By using the Reach function, the needs and accessibility of residents and tourists to share public service facilities are evaluated based on two aspects, facility availability and facility accessibility. Taking catering facilities as an example, the availability of facilities refers to calculating the number of residential areas, fishing village restaurants or hotels that can be served by catering facilities within a certain range. It reflects the supply of catering facilities. Considering the distribution and agglomeration of catering facilities, if the distribution of catering facilities is sparse and there are residential buildings that can serve residential buildings or fishing village restaurants and hotels, the value is extremely large. This indicates that the configuration of catering facilities in this area is lacking and should be increased. Suppose that certain catering facilities are concentrated and that there are few residential buildings or fishing village restaurants and hotels that can serve them. This indicates that there is redundant construction of catering facilities in the place and that the number of catering facilities should be reduced. Facility accessibility refers to the calculation of the number of catering facilities that can be reached within a certain range in residential areas, fishing village restaurants and hotels. It reflects the availability of catering facilities. The number of catering and shopping facilities that tourists and residents can reach within a certain range from the accommodation point is calculated, and whether the catering and shopping facilities in the study area focus on residents or tourists is evaluated in terms of the number of facilities that can be reached, reflecting group fairness. In addition, comparing the number of accessible facilities between regions can reflect the spatial fairness of facilities in different regions, making it possible to propose suggestions for the optimization of the facility space in the region.

## 3. Results

### 3.1. Spatial patterns of service facilities

**Figure 3:**
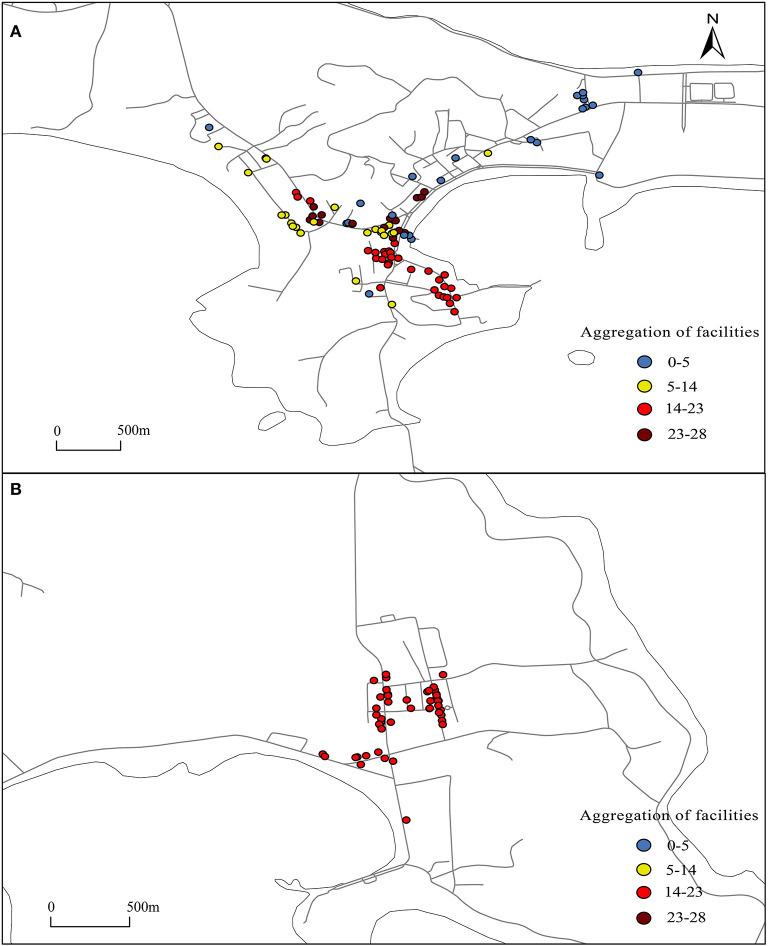
Facility agglomeration in the study area. **(A, B)** Setting both catering and shopping facilities as the starting and ending points, import the road data, and calculate the number of facilities that can be reached from other facilities within 800 meters. The greater the number of accessible facilities is, the higher the concentration of facilities.

The reach function of the UNA tool is used to analyze the agglomeration of facilities. Both catering and shopping facilities are set as the starting and ending points, road data are imported, and the number of facilities that can be reached from other facilities within 800 m is calculated. The greater the number of accessible facilities is, the higher the concentration of facilities. As shown in Figure 3A, the facilities in area A have a T-shaped distribution, and the facilities in area B have three clusters (3B). In general, the degree of agglomeration of facilities in area B is higher than that in area A (Figure 3). Dachangshan Island has areas with a lower degree of agglomeration of facilities, while the degree of agglomeration of facilities in the center of Guanglu Island is not much different.

### 3.2. Degree of facility availability

**Figure 4:**
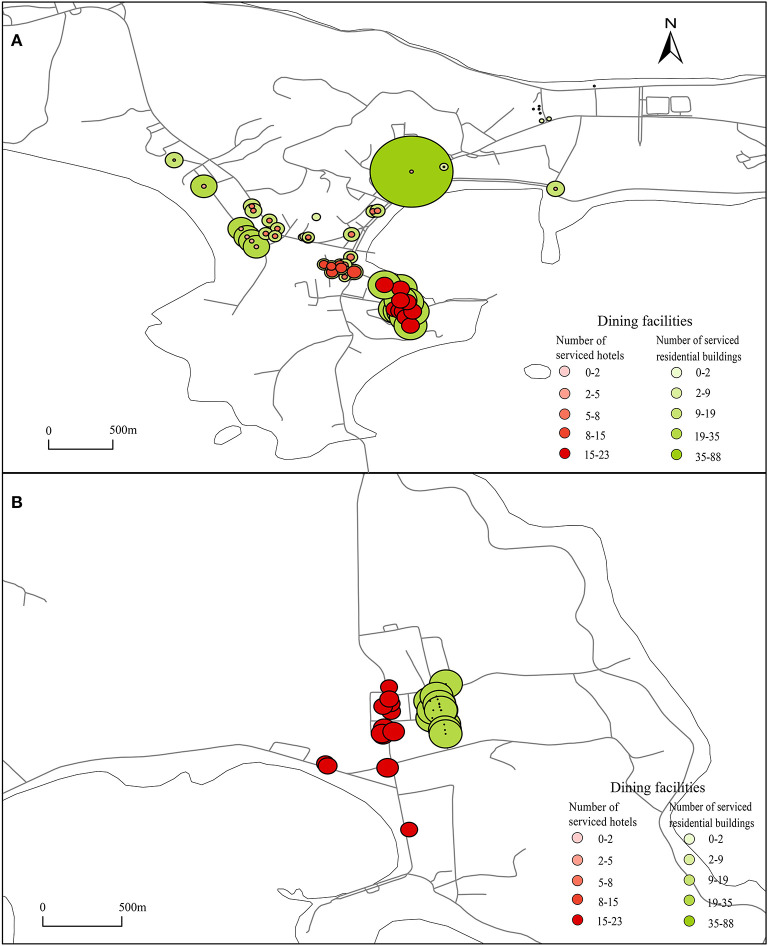
Dining facilities serving tourists. **(A, B)** Dining facilities were set as the starting point, fishing village restaurants, hotels and residential buildings were set as the ending point, import the road network, and calculate the reach index, then the reach index were assigned to each dining facilities.

**Figure 5:**
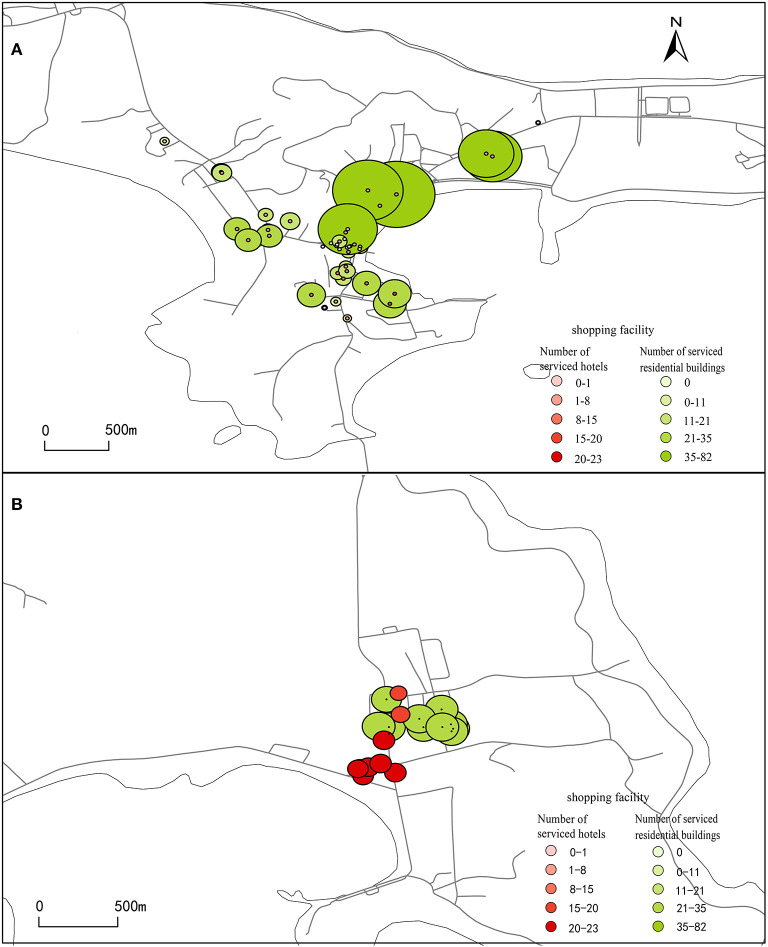
Shopping facilities serving tourists. **(A, B)** Shopping facilities were set as the starting point, fishing village restaurants, hotels and residential buildings were set as the ending point, import the road network, and calculate the reach index, then the reach index were assigned to each shopping facilities.

To analyze the utilization of dining facilities and shopping facilities in the overlapping area of visitor and resident activity spaces, catering facilities and shopping facilities were set as the starting point, tourist accommodation facilities such as fishing village restaurants and hotels and residential buildings were set as the ending point, the road network was imported, the reach index was calculated, and weights were assigned to each catering facility and shopping facility. The results are shown in Figures 4, 5.

#### 3.2.1. Degree to which catering facilities can be used by tourists

As shown in Figure 4A, within a range of 800 m, the catering facilities in area A that can be used by tourists from high to low: The People's Hospital of Changhai (about 19), the area from the south of Changhai Mall to the north of Changhai Market (within 6–15), the Sanpanian Community(within 5–6), the area from the People's Government of Dachangshan Island to Bilang Street (< 5), The latter two areas are considered to be unreasonably configured due to the large distribution of catering facilities but low availability. The area with the highest degree of utilization of catering facilities by other tourists' accommodation facilities in Guanglu Island Town Center is located between the Hongzhi Community and Jinhai Garden, with ~20 catering facilities (Figure 4B). The Nanda Line is a north-south main road that runs through the center of Guanglu Island town. The north side is mainly connected to Duoluomu Port. It is the main transportation line for tourists to enter and leave the island. There are many accommodation facilities along the line. Therefore, this area serves a small number of residents but a large number of tourists.

#### 3.2.2. Degree to which catering facilities can be used by residents

As shown in Figure 4A, the catering facilities can be used by residents within 800 m from high to low: the People's Government of Dachangshan Island (Labaichuan Store, can serve for 88 residential building), the People's Hospital of Changhai County (about 35), the Sanpanian Community, (within 14–28), the area between Changhai Mall and Changhai Market (within 9–19), the area from the People's Government of Dachangshan Island to Bilang Street (< 9). First, the point with the highest degree of utilization is Dongshan District on the north side. The number of residential buildings near the store is high, but the number of dining facilities is minimal. This finding indicates that the area where the point located is a catering facility demand area and that catering facilities can be appropriately configured to provide services for residents and relieve the pressure of catering demand at this point. Secondly, the area with the highest degree of utilization, that is, the area where catering facilities can be utilized by tourists. The catering facilities in this area, whether for tourists or residents, are the highest degree of utilization, indicating that catering facilities are reasonably configured. Finally, there are two areas where catering facilities are constructed repeatedly, that is, the number of catering facilities is large, but the degree of serving tourists and residents is low. There are ~35 catering facilities in the center of Guanglu Island that can serve tourists. The catering facilities between residential buildings can be highly utilized by residents; the catering facilities along the road can be highly utilized by tourists (Figure 4B).

#### 3.2.3. Degree to which shopping facilities can be used by tourists

As shown in Figure 5A, the shopping facilities in the area southeast of the People's Hospital of Changhai have the highest level of service for tourists. Additionally, this area is also the area with the highest level of catering facilities serving tourists. This finding shows that tourists can easily get catering services and shopping services here. Secondly, the Changhai Mall is the area with the most concentrated shopping facilities in Dachangshan Island, but the availability of facilities is low, have redundant construction of shopping facilities when only serving tourists. Finally, the shopping facilities in the areas east, west and north of Changhai Mall serve tourists less well, because there are fewer shopping facilities. The area with the highest degree of utilization of shopping facilities in Guanglu Island Center is located near the intersection of the Nanda Line and Dongxu Line (Figure 5B). This area can serve 23 hotels, while a single shopping facility on Dachangshan Island can serve up to 19 hotels.

#### 3.2.4. Degree to which shopping facilities can be used by residents

Through the analysis of Figure 5A: First, the public service facilities in two regions are unreasonable. One region has too many facilities and is idle, while the other region has insufficient facilities. Second, there is still an area with reasonable allocation of public service facilities. he shopping facilities in area A can serve up to 82 residential buildings, which is close to the maximum number of residential buildings served by catering facilities. Additionally, It is also located near the People's Government of Changhai County. It means that there is a greater demand for dining facilities and shopping facilities. The shopping facilities in the Changhai Mall are not highly usable by tourists and residents, and there is redundant construction of catering facilities and shopping facilities. However, the People's Government of Changhai County have high demand for catering facilities and shopping facilities. Thus, the shopping and catering facilities near Changhai Mall can be transferred to this area. The vicinity of the People's Hospital of Changhai is an area with a large number of catering and shopping facilities serving residents and tourists. Not only can provide catering and shopping services for tourists during the tourist season but also can service residents during the tourist off-season. It has become an area with a reasonable configuration of facilities in the center of Dachangshan Island. Figure 5B shows that the shopping facilities in Guangludao Center can be most utilized by residents is located between the buildings.

### 3.3. Facilities accessible to residents and tourists

**Figure 6:**
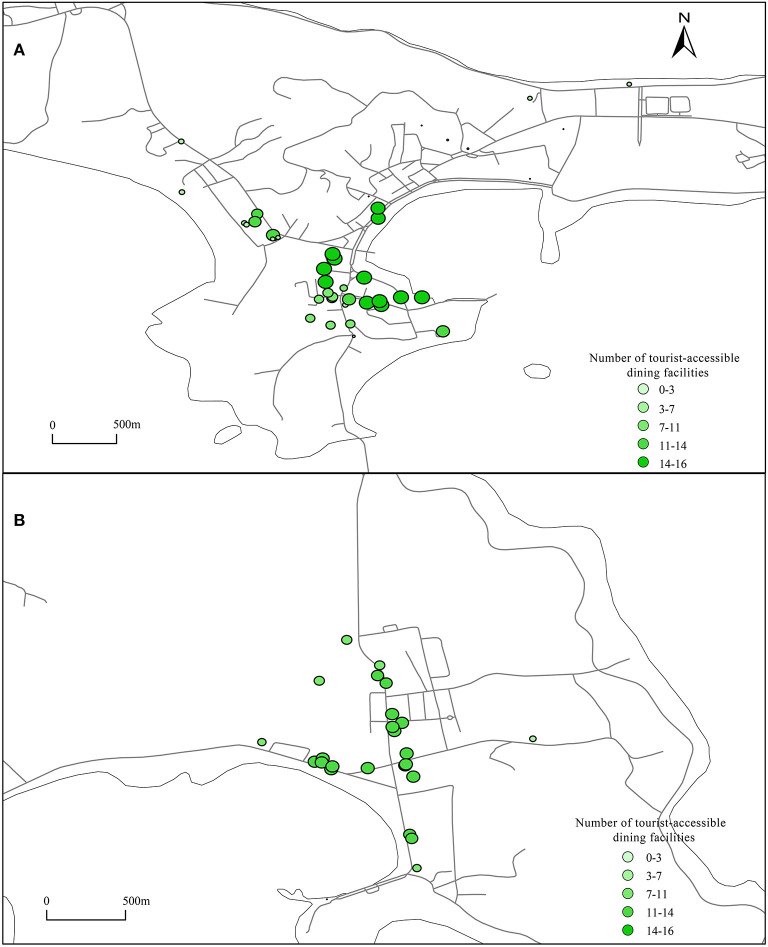
Catering facilities accessible to tourists. **(A, B)** Using the UNA analysis tool, set the tourist accommodation facilities as the starting point, catering facilities as the ending point, import the road network, and calculate the number of catering facilities that can be reached from the tourist accommodations within a range of 800 meters.

**Figure 7:**
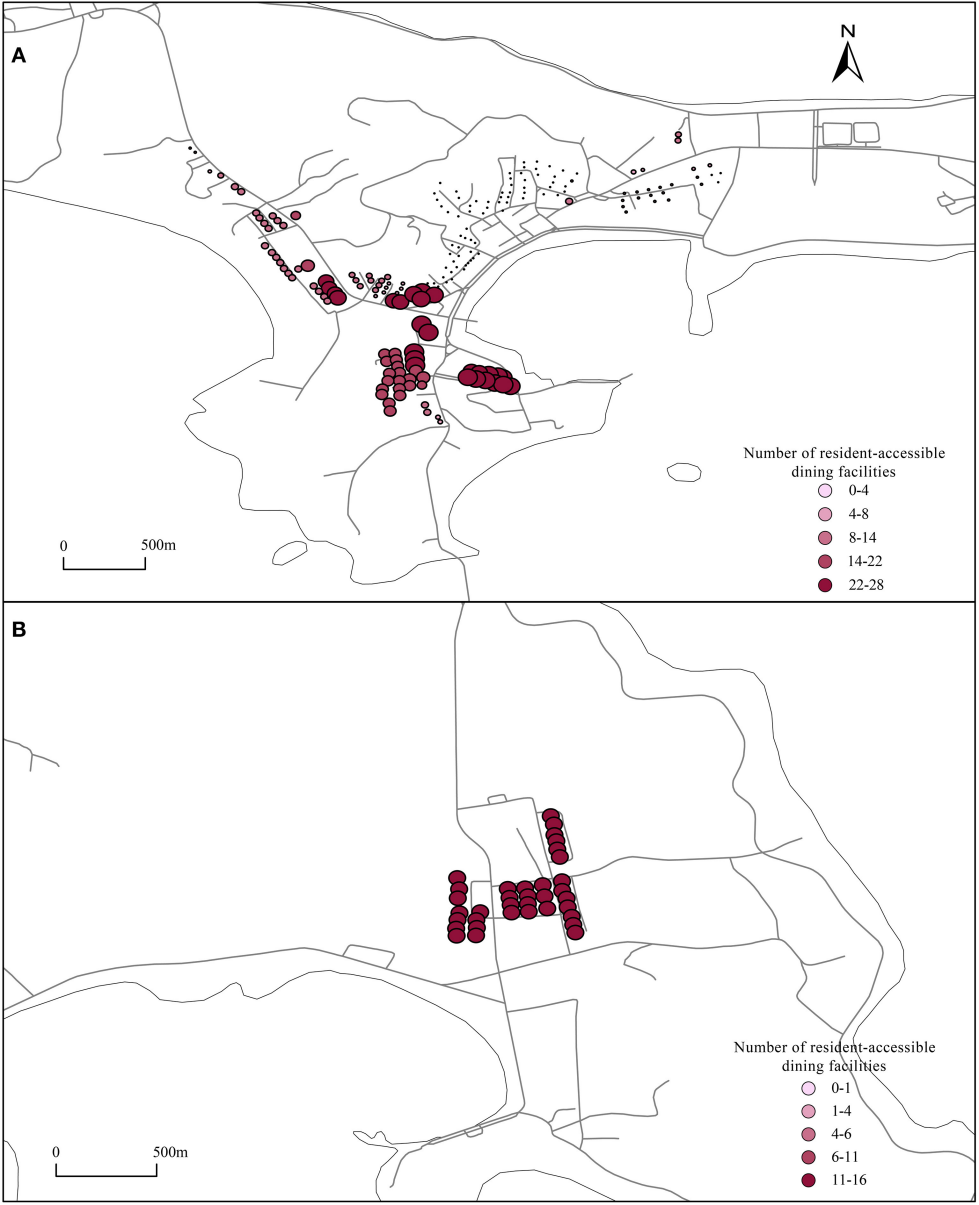
Catering facilities accessible to residents. **(A, B)** Using the UNA analysis tool, set the residential buildings as the starting point, catering facilities as the ending point, import the road network, and calculate the number of catering facilities that can be reached from residential buildings within a range of 800 meters.

**Figure 8:**
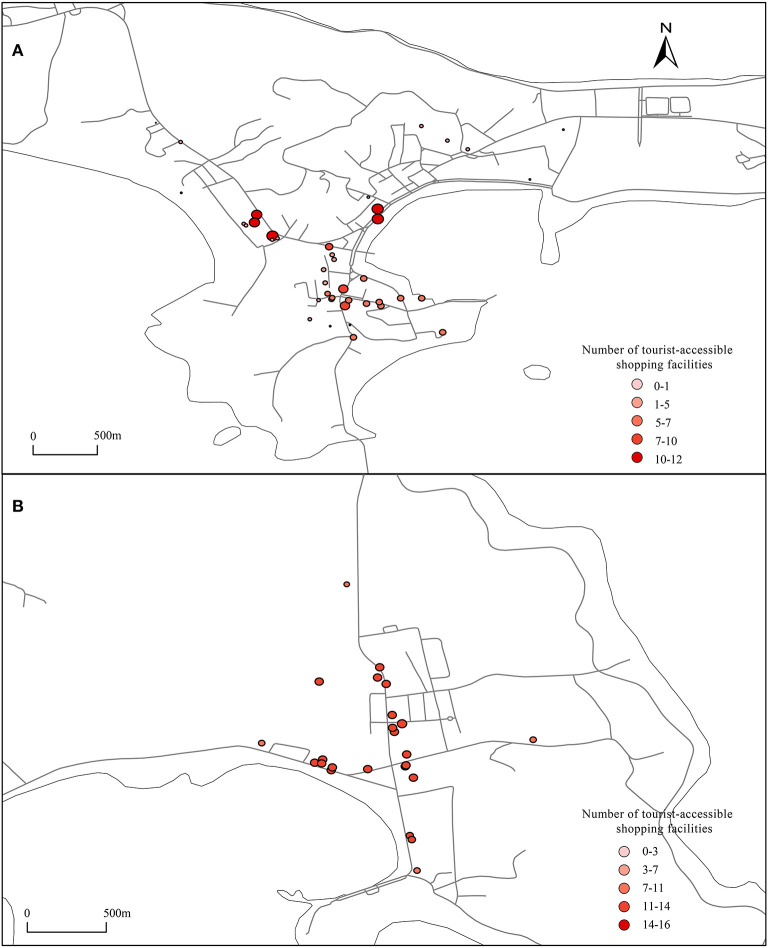
Shopping facilities accessible to tourists. **(A, B)** Using the UNA analysis tool, set the tourist accommodation facilities as the starting point, shopping facilities as the ending point, import the road network, and calculate the number of shopping facilities that can be reached from tourist accommodations within a range of 800 meters.

**Figure 9:**
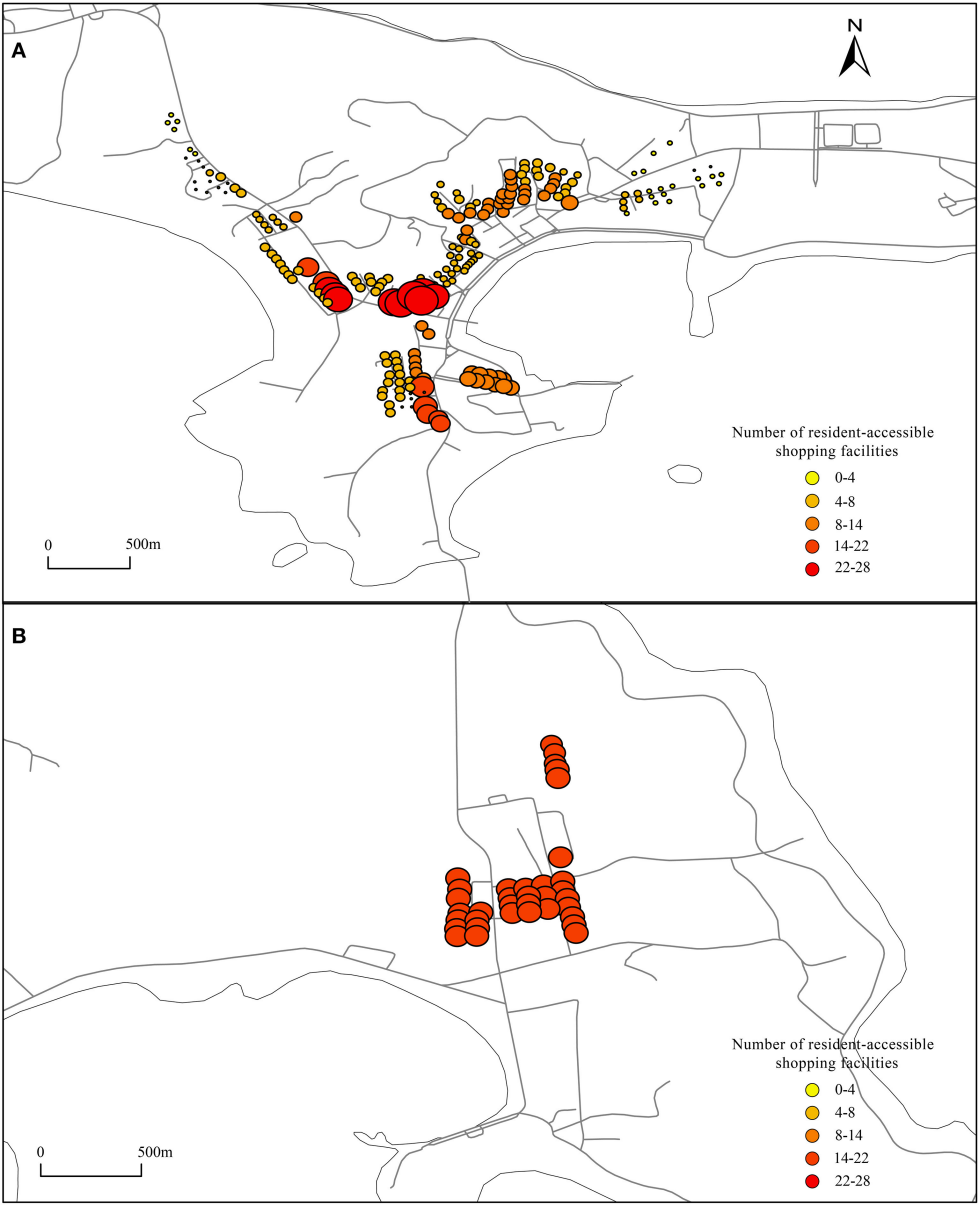
Shopping facilities accessible to residents. **(A, B)** Using the UNA analysis tool, set the residential buildings as the starting point, shopping facilities as the ending point, import the road network, and calculate the number of shopping facilities that can be reached from residential buildings within a range of 800 meters.

Using the UNA analysis tool, tourist accommodation facilities and residential buildings were set as the starting point, dining facilities and shopping facilities were set as the ending point, and then, the road network was imported. The number of shopping and dining facilities that can be reached from residential buildings and tourist accommodations within a range of 800 m was calculated, and the results are shown in Figures 6–**9**.

#### 3.3.1. Catering facilities accessible to tourists

As shown in Figure 6A, the areas where tourists in area A can reach the highest number of catering facilities are the area between the Changhai Shopping Center and Changhai Market as well as the area southeast of the People's Hospital of Changhai. The number of accessible catering facilities is ~16, which is the same as the number of catering facilities accessible to residents in this area. This finding indicates that the configuration of catering facilities in this area can reflect group fairness. The points where tourists can reach the most catering facilities within 800 m of Guanglu Island Town Center are located along the Nanda Line and Dongxu Line, and the number of accessible catering facilities is between 11 and 14 (Figure 6B). Combined with the facilities accessible to residents in Figure 7B, the number of catering facilities accessible to residential buildings is 14. This finding indicates that the layout of catering facilities in the center of Guangludao town is relatively fair and slightly biased toward residents.

#### 3.3.2. Catering facilities accessible to residents

As shown in Figure 7A, on the east side of the People's Hospital of Changhai, there are a large number of catering facilities accessible to residential buildings, and the catering facilities can be used by residential buildings to a high degree. For residential buildings such as Sanpanian Community, the number of catering facilities available within 800 m is lower, but there are more residential buildings. Within 800 m, the number of catering facilities that residents can reach is small, the number of services that a single facility can serve is as high as 88, and facilities are in short supply. From the Figure 7B, the number of catering facilities within 800 m of each residential building on Guanglu Island is 14, indicating that the layout of catering facilities in the center of Guanglu Island is fair for each residential building.

#### 3.3.3. Shopping facilities accessible to tourists

**Figure 9A** shows that the configuration of shopping facilities in the center of Dachangshan Island is strongly biased toward residents. The maximum number of shopping facilities that residents can walk to and reach is 16, but the maximum number of shopping facilities that tourists can reach is only 12 (Figure 8A), located in Sanpanian Community. In addition, the number of accessible shopping facilities in the area south of Changhai Mall is ~7. That means there is some kind of obstacle in the area that hinders passage, resulting in a decrease in the number of accessible facilities. Visitors to Guanglu Island have fewer accessible shopping facilities than dining facilities. The number of accessible dining facilities is ~14 (Figure 6B), and the number of accessible shopping facilities is ~9 (Figure 8B). These findings indicate that there is little difference in the number of dining and shopping facilities available to tourists.

#### 3.3.4. Shopping facilities accessible to residents

As shown in Figure 9A, some residential buildings in the Sanpanian Community have 24-26 shopping facilities within 800 m. At the same time, the centralized distribution of shopping facilities also affects the number of shopping facilities accessible to residential buildings in the area south of Changhai Mall, reaching between 8 and 20. There is a large difference in the number of accessible dining facilities and shopping facilities in the Dongshan Community. The number of catering facilities accessible to the residential buildings in this area is ~1, but the number of accessible shopping facilities is between 6 and 14, indicating that there are redundant shopping and catering facilities on the east and west sides that should be appropriately transferred to this area to meet the needs of residents. As shown in Figure 9B, a single residential building in the center of Guanglu Island has 14 accessible catering facilities and ~20 accessible shopping facilities. The number of accessible shopping facilities is higher than the number of accessible catering facilities. The reason is residents mainly eat at home; thus, their daily shopping needs are slightly higher than their catering needs, indicating that in terms of the configuration, the ratio of catering facilities to shopping facilities in the study area is in line with the actual situation.

## 4. Discussion and conclusion

### 4.1. Discussion

In this paper, the spatial and temporal behavior of differentiated groups is used to delineate the overlapping area of the behavioral space. Selecting catering and shopping facilities with a high degree of sharing and evaluating the configuration of facilities from the perspective of accessibility and availability hold practical significance for the optimal configuration of island public service facilities. In the tourist off-season, residents constitute the main activity group, and in the peak tourist season, there are a large number of tourists. The facility configuration in the overlapping area of the behavioral space needs to consider not only the issue of group fairness but also the issue of configuration efficiency. This paper analyzes the accessibility of public service facilities in the context of multiple groups of tourist destinations.

(1) Based on the availability of facilities (Table 1), there is a serious mismatch between the spatial distribution of facilities and residential places in the study area. This mismatch includes not only the mismatch in the spatial distribution of shopping and dining facilities and tourist residential places but also the dislocation in the spatial distribution of residential buildings. Due to the locationally inappropriate distribution of facilities and accommodation, some areas have redundant construction of facilities or a lack of configuration. Differently, there are few catering and shopping facilities near the People's Government of Changhai County, but the availability of catering and shopping facilities for residents is high. These findings indicate that the configuration of shopping and catering facilities here is unreasonable for residents and should be increased. At the same time, the degree of utilization by tourists is low, which shows that the configuration of catering and shopping facilities has little impact on tourists and that the configuration of facilities here can consider only the needs of residents.

**Table 1:** Facility configuration in the study area.

**Facilities**	**Utilization by tourists**	**Utilization by residents**	**Number**	**Evaluation**	**Location**
Catering facilities	High		Many	Reasonable	The area southeast of the People's Hospital of Changhai Qingfeng Street, Sanpanian Community
	Low		Many	Too much	The area southeast of the People's Hospital of Changhai Qingfeng Street, Sanpanian Community
		High	Many	Reasonable	The area southeast of the People's Hospital of Changhai
		High	Few	Not enough	The area near the the People's Government of Changhai
		Low	Many	Too much	Qingfeng Street, Sanpanian Community, etc.
Shopping facilities	High		Many	Reasonable	The area southeast of the People's Hospital of Changhai
	Low		Many	Too much	The Changhai Mall, Sanpanian Community
		High	Many	Reasonable	The area southeast of the People's Hospital of Changhai
		High	Few	Not enough	The area near the the People's Government of Changhai
		Low	Many	Too much	The Changhai Mall

(2) Based on the accessibility of facilities, the configuration of catering and shopping facilities in the study area generally shows a tendency to serve residents, with more facilities accessible to residents than to tourists. The main reason is that these facilities serve residents over the course of the whole year and serve tourists only in the peak tourist season. However, in some areas, the configuration of catering facilities is fair to tourists and residents and can reflect group equity. These facilities are mainly distributed in the area southeast of the People's Hospital of Changhai. The number of catering facilities accessible to tourists and residents is ~16, and the degree of sharing of catering facilities is high.

Except for those areas with a reasonable configuration of catering and shopping facilities, the facilities in other areas should be configured in areas with high accessibility for residents and tourists as much as possible to reduce the spatial dislocation between facilities and residential areas for residents and tourists, improve the degree of sharing and accessibility of facilities, avoid the waste of facilities in the tourist off-season, and improve the efficiency of facility configuration. Taking areas with excessive facility construction as an example, their catering or shopping facilities should be transferred to areas with few facilities as much as possible to improve the utilization efficiency of facilities. In addition, if there are few catering and shopping facilities accessible to residents and tourists in a certain area but there are many residential buildings and there are few hotels, then even though the configuration of facilities does not conform to the principle of a high degree of sharing, since the configuration of facilities is of little significance to tourists, the needs of residents should mainly be considered, and appropriate new facilities should be added.

Carrying out an evaluation of the facility configuration from the microlevel perspective of individuals' spatiotemporal behavior is meaningful for exploring human-oriented spatial planning under the background of changing activity groups in the future space. However, this paper has the following shortcomings: Firstly, the public service facilities studied only include catering and shopping facilities, without considering other types of public service facilities, due to the limitations of the data acquired, the public service facility data that can be obtained is greatly limited. Secondly, this paper only considers the number of facilities, not the scale of facilities. In the future, we can obtain more detailed public service facility data and population data for in-depth research. Finally, the accessibility of facilities only considers the spatial accessibility, while the time accessibility is not considered in this manuscript. In fact, since public service facilities are not open all day, accessibility will be affected by the opening time of public service facilities, resulting in space accessibility but time unreachability.

### 4.2. Conclusion

The theory of temporal geography holds that the existence of individuals in spatial places involves the consumption of time and space. Differences in the purpose of the temporal and spatial behavior of different groups lead to differences in their utilization of the same space. The infrastructure in a space is required to provide diversified services to meet the needs of different groups. Due to the development of tourism, destinations no longer are defined by a single main activity. At the same time, meeting the tourism needs of tourists and the living needs of residents has posed new challenges to the optimization of the space of public service facilities. However, to achieve group equity as much as possible, the allocation of public service facilities needs to undergo through different stages. Therefore, this paper takes a tourist island with obvious differences in the main active groups in the same space as an example and delimits the study area by the characteristics of the temporal and spatial behavior of different groups, that is, the overlapping area of the behavioral space of different groups. Based on the two aspects of facility accessibility and availability, research evaluating the configuration of catering facilities and shopping facilities in the study area is conducted, and the results of the study are as follows.

(1)The “dislocation” of spatial distribution between public service facilities and residential areas is serious, and only one regional facility configuration is reasonable. It is suggested that the public service facilities that can be used at a low level should be moved to the areas with large demand for facilities, so as to improve the utilization efficiency of public service facilities and reduce the “dislocation” of space between residential areas and facilities.

(2)The number of facilities accessible to residents on Dachangshan Island and Guanglu Island is generally greater than that accessible to tourists. The public facilities serve for residents more than the tourists is a right choose. Because the residents are the foundation of the island development, but the tourists just an important outside factors, it's a driving force to the development of the island, it is also important, but not the first important. Guaranteeing the daily needs of islanders and meeting the diverse tourism needs of tourists are the basis for the sustainable development of island tourism.

This paper analyzes the accessibility of public service facilities in the context of multiple groups of tourist destinations and is an active exploration of the configuration of public service facilities under the background of changing activity groups in the future space.

## Data availability statement

The original contributions presented in the study are included in the article/supplementary material, further inquiries can be directed to the corresponding author.

## Author contributions

XZ was responsible for the writing of the manuscript. DZ provided the writing ideas of the paper and gave guidance in the writing process. All authors contributed to the article and approved the submitted version.
